# Structure and assembly process of fungal communities in the Yangtze River Estuary

**DOI:** 10.3389/fmicb.2023.1220239

**Published:** 2024-01-08

**Authors:** Wu Qu, Yaqiang Zuo, Yixuan Zhang, Jianxin Wang

**Affiliations:** Marine Science and Technology College, Zhejiang Ocean University, Zhoushan, China

**Keywords:** fungal community, stochastic process, community assembly, Yangtze River Estuary, keystone taxa

## Abstract

Marine fungi are essential for the ecological function of estuarine ecosystems. However, limited studies have reported on the structure and assembly pattern of the fungal communities in estuaries. The purpose of this study is to reveal the structure and the ecological process of the fungal community in the Yangtze River Estuary (YRE) by using the amplicon sequencing method. Phyla of Ascomycota, Basidiomycota, and Chytridiomycota were dominant in the seawater and sediment samples from YRE. The null model analysis, community-neutral community model (NCM), and phylogenetic normalized stochasticity ratio (pNST) showed that the stochastic process dominated the assembly of fungal communities in YRE. Drift and homogeneous dispersal were the predominant stochastic processes for the fungal community assembly in seawater and sediment samples, respectively. The co-occurrence network analysis showed that fungal communities were more complex and closely connected in the sediment than in the seawater samples. Phyla Ascomycota, Basidiomycota, and Mucoromycota were the potential keystone taxa in the network. These findings demonstrated the importance of stochastic processes for the fungal community assembly, thereby widening our knowledge of the community structure and dynamics of fungi for future study and utilization in the YRE ecosystem.

## Highlights

- Stochastic processes dominated the assembly of fungal communities in the Yangtze River Estuary (YRE).
- The fungal communities in YRE showed a distance–decay pattern.
- Ascomycota, Basidiomycota, and Mucoromycota were the keystone taxa for the fungal networks.

## Introduction

Estuaries, which are located at the interface between land and ocean, provide high ecological productivity, goods, and services to humans (Beck et al., 2001), and this particular ecological location is often used as the nursery, sanctuary, and growth areas for many species, thereby playing considerable roles in food webs and energy flux (Chevillot et al., 2019). The Yangtze River Estuary (YRE) and the adjacent East China Sea have an effective and particular function in the mutual interactions of the land and sea life in East Asia (Zhang J. et al., 2016). In the YRE, material inputs from terrestrial and marine sources are mixed by the hydrodynamic processes, and the resulting distributions have important implications for regulating food web structure and ecosystem function (Zhang J. et al., 2016).

Fungi are an integral part of marine ecosystems and can exist in almost all the explored marine habitats, from the ocean surface to the deep sea (Amend et al., 2019), such as seawater columns (Taylor and Cunliffe, 2016), sediments (Orsi et al., 2013), and estuaries (Mohamed and Martiny, 2011). Diverse fungi are detected in the estuary ecosystem (e.g., Ascomycota, Basidiomycota, Glomeromycota, and Chytridiomycota; Mohamed and Martiny, 2011), and these communities of estuarine fungi contribute to the element cycles, biological carbon pumps, commensal enzymes, and bio-pathogenicity in estuaries due to their special properties (Amend et al., 2019). Hence, the knowledge of fungal communities is of great significance for understanding the ecological functions of estuary ecosystems, including carbon sink, resource development, and biological interaction (Tsui and Hyde, 2004).

The study on the community assembly is a crucial component of the research on fungal communities. The ecological processes, including deterministic and stochastic, are crucial to explain the assembly of microbial communities (Logares et al., 2013; Nemergut et al., 2013b; Isabwe et al., 2022). The deterministic processes is are primarily interactions between biotic and abiotic factors, such as the interactions that exist between species (e.g., competition, predation, mutualism, and tradeoff) and environmental filtering (e.g., salinity, pH, temperature), which together shape community compositions (Chesson, 2000; Fargione et al., 2003), stochastic processes that consider all species as ecologically equivalent, and community structures that are shaped by random factors, such as random births, deaths, dispersal, extinction, and speciation (Chave, 2004; Hubbell, 2011).

The environmental DNA metabarcoding technology typically includes the 18S rRNA (Zuo et al., 2022), ITS (Yu et al., 2022), and 16S rRNA (Huang Y. et al., 2022) amplicons by using special primers. This technology has been widely used in studies on the assembly mechanisms of bio-communities with the development of high-throughput sequencing. For instance, previous studies have shown that stochastic processes have a strong effect on bacterial community assembly in Yellow River Estuary (Huang L. et al., 2022), Pearl River Estuary (Wu et al., 2020), and YRE (Shi et al., 2022). Therefore, stochastic processes might also impact the fungal community assembly in the YRE; however, this hypothesis has not been tested until now. The current study aims to assess the importance of the stochastic processes for the assembly of fungal communities in the YRE by using the 18S rRNA amplicon sequencing method, thereby elucidating the dynamics of the fungal communities in the YRE.

## Materials and methods

### Sample collection

**Figure 1:**
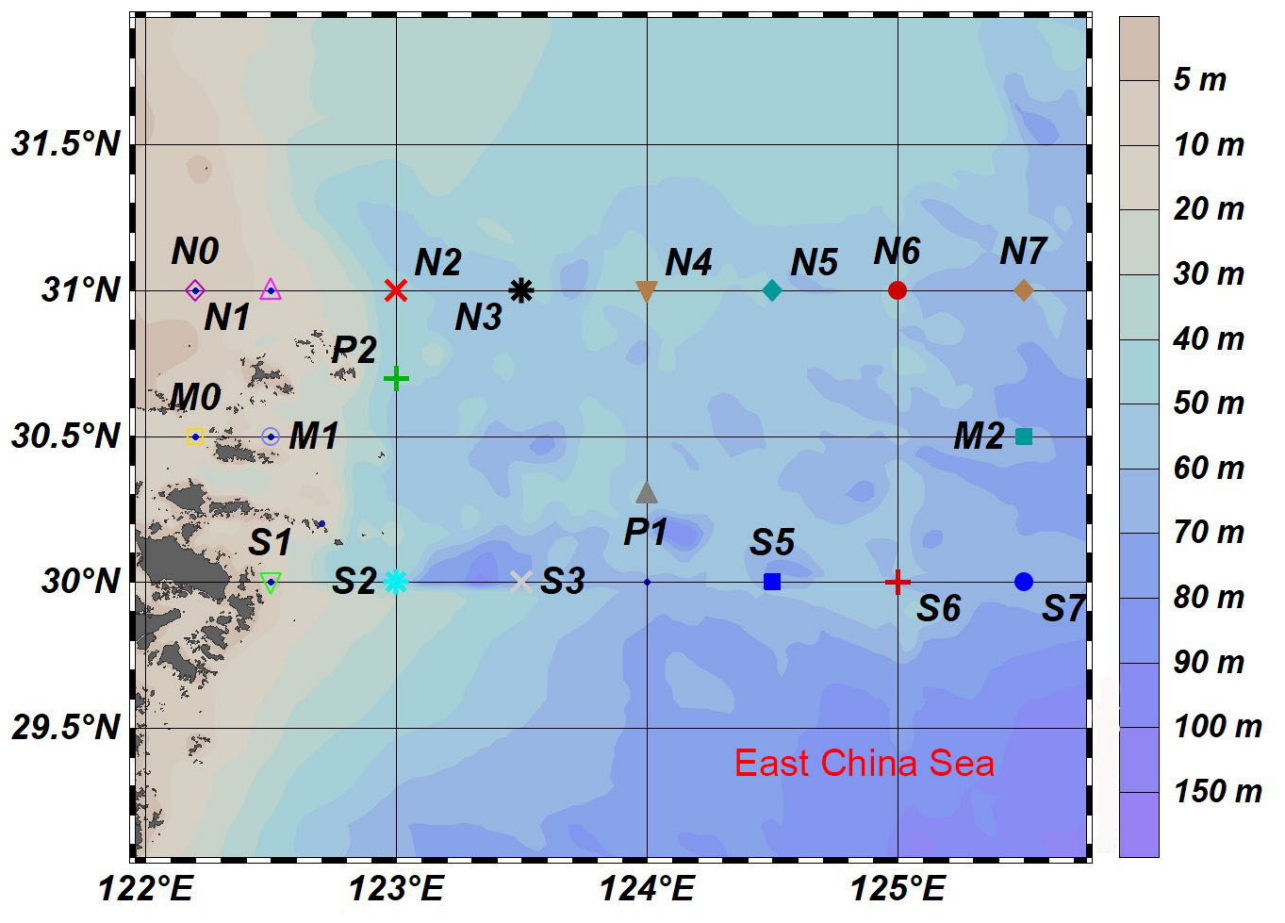
The sampling sites of this study in the Yangtze River Estuary (YRE).

This study was carried out in the East China Sea across the YRE and its adjacent waters (125°–126°E, 30°–31°N) in August 2020 (Figure 1). Water samples were collected from the surface, middle, and bottom layers at 21 stations by using an SBE32 CTD (Washington, Sea-Bird Electronics). Seawater was pre-filtered through the 3.0-μm-pore size polycarbonate membranes and then was filtered through the 0.22-μm-pore size polycarbonate membranes (47 mm diameter; Millipore, Germany; Wu et al., 2020). Sediment samples were taken directly from the topsoil of each site. Each water sample was named with the location name, followed by the abbreviation of the layer and the membranes, and each sediment sample was named by the site. The water samples were divided into three groups: surface, middle, and bottom. For example, the M0SP sample means the water sample in the surface seawater at the M0 site. The M0 sample means the sediment sample collected at the M0 site.

### Measurement of the environmental factors

The ammonia nitrogen (NH_4_-N), nitrite nitrogen (NO_2_-N), and phosphorus (PO_4_-P) of seawater were determined by SmartChem automatic nutrient analyzer (Seabird Company, Washington). The nitrate (NO_3_-N) of seawater was manually analyzed and determined by the Cu–Cd reduction method (del Carmen et al., 2006). The analysis of the pH of seawater was conducted using a pH meter (Leici, Shanghai). The sediment pH, NH_4_-N, NO_2_-N, NO_3_-N, and PO4-P were measured by Nanjing Yanke Testing Technology Co., Ltd. (Nanjing, China).

### Extraction of environmental DNA and sequencing of 18S rRNA genes

The DNA in samples was extracted using a FastDNA Spin Kit (MP Biomedicals, United States) according to the instructions. The integrity and quality of DNA were measured by 1% agarose gel, and the DNA concentration and purity were verified using a NanoDrop 2000 UV–vis spectrophotometer (Thermo Scientific, Wilmington, United States). Primer pairs of SSU0817F (5′-TTAGCATGGAATAATRRAATAGGA-3′) and 1196R (5′-TCTGGACCTGGTGAGTTTCC-3′; Zheng et al., 2020) were used to amplify the 18S rRNA genes from the DNA solution by using the ABI GeneAmp 9700 PCR thermocycler (ABI, CA, United states). The sequencing was conducted using the Illumina Miseq PE300 platform/NovaSeq PE250 platform (Illumina, San Diego, United States) by Majorbio Bio-Pharm Technology Co. Ltd. (Shanghai, China). Raw sequence data are deposited at the NCBI Sequence Read Archive under the accession numbers SRR21079128–SRR21079264.

### Sequence processing and fungal community analysis

The VSEARCH v.2.15.1 was used to perform the merge of the pair-end FASTQ sequences (Rognes et al., 2016). The *fastx_filter* command was used to delete the tags and primers of the original sequence, and the error rate was controlled to be less than 0.01. The *Derep_fulllength* command was used for the de-redundancy of the sequence, and the unoise3 feature in the USEARCH tool was used to de-noise in order to obtain single-base accuracy amplicon sequence variants (ASV; Edgar, 2010). Then, the sequences were de-chimerized using VSEARCH and the Silva database (SILVA_18s_v138.fa). Then, the fungi taxonomy of each ASV representative sequence was analyzed using SINA version 1.2.11 against the Silva 138 database at a confidence threshold of 0.7 (Pruesse et al., 2012). Finally, 280 high-quality sequences of fungi were obtained from 137 samples.

The correlations between environmental factors and alpha-diversity indices were calculated using the “corrplot” package of R software. Diversity indices, including Shannon and Chao1, were calculated using the “picante” package of R software (Kembel et al., 2010) and were visualized using the “ggpubr” package of R software. A one-way analysis of variance (ANOVA) was used for multiple-group comparison of the data. Heatmaps were visualized using R packages of “psych” (Revelle and Revelle, 2015). The environmental factor with variance inflation factor value of <10, calculated using the function vif() in the “car” package of R (Fox et al., 2007), was selected for the linear regression to avoid the multilinearity among the factors (Craney and Surles, 2002; Abadura et al., 2015). The importance of each environmental variable for the community was assessed with the multiple linear regression (Jiao et al., 2020) using calc.relimp() in the relaimpo package of R (Grömping, 2007). R package of “ggalluvial” (Brunson, 2020) was used for visualizing the fungal community composition at the phylum level. The constrained principal coordinate analysis (CPCoA) was used to reveal the fungal community using the package “amplicon” of R software (Liu et al., 2021). An unweighted pair-group method with arithmetic means (UPGMA) clustering analysis was performed to display the dissimilarity among different groups based on the Jaccard dissimilarity index, Bray–Curtis dissimilarity index, Euclidean dissimilarity index, and Manhattan dissimilarity index using “vegan” package of R software (Oksanen et al., 2007).

Beta-diversity of the samples was partitioned into richness difference components (Sørensen dissimilarities) and replaced using the packages “BAT” (Cardoso et al., 2014) and “betapart” of R software (Baselga and Orme, 2012; Si et al., 2017). The de-trend correspondence analysis (DCA) was performed using decorana() of the vegan package of R software. Based on the gradient length along the axis (<4) of the DCA result, the distance-based redundancy analysis (db-RDA) was used to fit the fungal community matrix with the environmental variables using the “vegan” package (Oksanen et al., 2007) to reveal their relationships.

### Assembly process analysis of fungal community

Community similarity was used to express the relationship between community dissimilarity and geographic distance. The geographic distances between different samples based on the latitude and longitude coordinates were calculated using the “vegdist” function in the R “vegan” (Oksanen et al., 2007) package. For both latitudinal diversity gradient and distance decay relationships, a linear regression analysis was carried out to calculate the slope, *R*-value, and significance values. A neutral community model (NCM) was used to predict the potential importance of stochastic processes in fungal assembly by determining the relationships between the detection frequency of fungal taxa in a set of local communities (Sloan et al., 2006; Chen et al., 2019) using the minpack.lm (Elzhov et al., 2016) and HMisc (Harrell and Harrell, 2019) packages.

The fungal community assembly patterns including stochastic and deterministic processes (Zhang et al., 2021) were determined as follows: the beta nearest-taxon index (βNTI) was calculated based on the phylogenetic distance and ASV abundances, which indicated the number of the deviation degree of the beta mean nearest taxon distance (βMNTD) of the null model developed by Stegen et al. (2013). Bray–Curtis-based Raup-Crick (RCBray) and βNTI were used to assess the ecological processes. The |βNTI| ≥ 2 indicated that deterministic processes including homogeneous selection (the βNTI value < −2) and variable selection (the βNTI value > 2; Stegen et al., 2012; Zhou and Ning, 2017) played a more significant role than the stochastic process, whereas |βNTI| < 2 represented a more important role of stochastic processes. Furthermore, |βNTI| < 2 and |RCBray| < −0.95 showed that homogenizing dispersal dominated the fungal community assembly. |βNTI| < 2 and |RCBray| > 0.95 indicated a crucial impact of dispersal limitation, and |βNTI| < 2 and |RCBray| < 0.95 suggested the significant role of the drift. The βNTI and RCBray values were calculated by R script “bNTI_Local_Machine.r” (Stegen et al., 2012). The phylogenetic normalized stochasticity ratio (pNST) was used to quantify the relative importance of deterministic and stochastic processes in community assembly based on the phylogenetic beta diversity index and phylogenetic randomization. pNST < 0.5 indicates a significant role in deterministic processes, while pNST > 0.5 indicates that stochastic processes dominated the community assembly (Ning et al., 2019).

### Co-occurrence network analysis

The rcorr() function of R software was used to perform the pairwise correlations based on ASV relative abundance and the R package “Hmisc” was used to build the co-occurrence network of the microbial community (Harrell and Harrell, 2019). The false discovery rate correction was conducted to adjust the *p-*value. The ASVs with an R^2^ value > 0.7 and a *p-*value < 0.05 remained for the network. The R package of “igraph” (Csardi and Nepusz, 2006) and Gephi v0.92 (Bastian and Heymann, 2009) were used to build and visualize the network diagram, respectively. The calculation of topology characteristics of the community networks (including degree, clustering coefficient, and average path length) and the analysis of the network modules were also performed using Gephi v0.92 with default parameters. The ASVs with Zi ≥ 2.5 and/or Pi ≥ 0.62 in the networks were identified as the keystone taxa (Deng et al., 2012).

## Results

### Environmental parameters

Environmental properties in the current study are listed in Supplementary Table S1. Among these factors, NH_4_-N was significantly and positively correlated with PO_4_-P, and NO_2_-N (*p* < 0.01) and was negatively correlated with NO_3_-N and pH (*p* < 0.01). In addition, NO_2_-N and PO_4_-P was negatively correlated with NO_3_-N (*p* < 0.01; Supplementary Figure S1).

### Community composition

**Figure 2:**
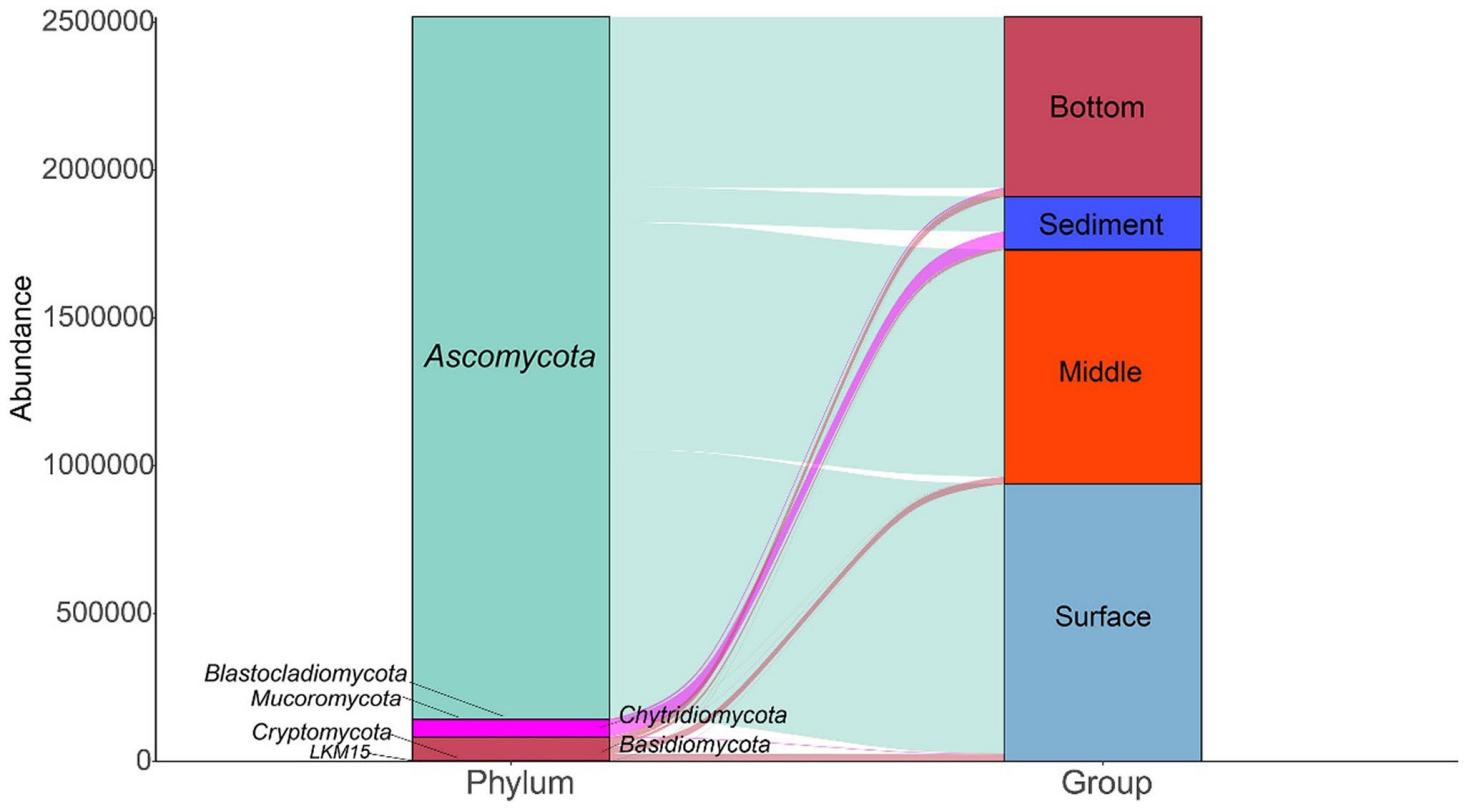
The fungal community compositions at the phylum level in the seawater and sediment samples.

A total of 7 phyla were annotated in 137 samples (Figure 2). Ascomycota was the most abundant phylum in all samples, followed by Basidiomycota and Chytridiomycota. The relative abundance of Ascomycota was higher in the surface layer of seawater than in the middle layer and bottom layer of the seawater and sediment. In addition, the relative abundance of Chytridiomycota was higher in the sediment layer than in the different layers of seawater.

### Alpha- and beta diversity of the fungal communities

**Figure 3:**
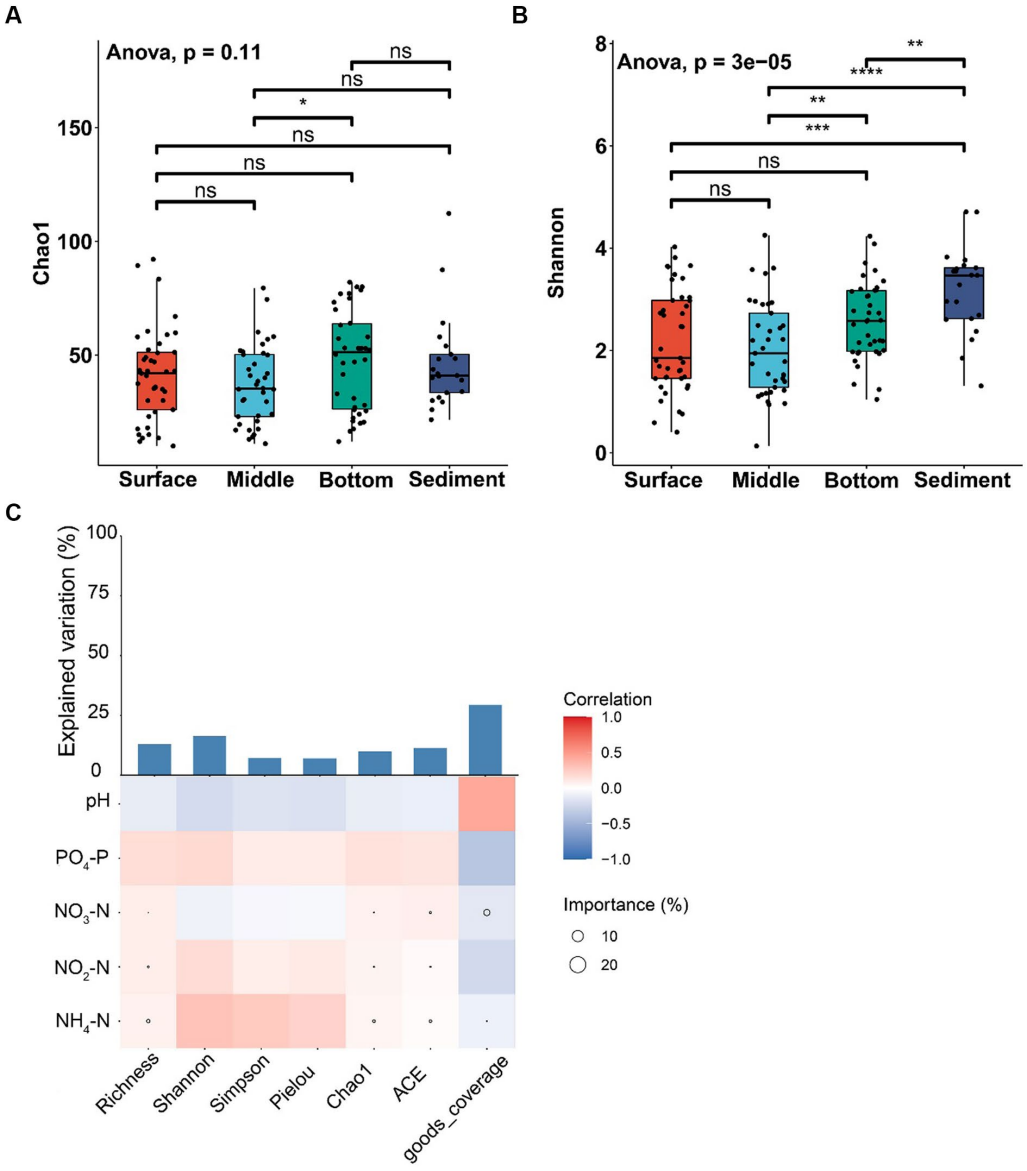
Alpha-diversity analysis of fungal communities among the samples from different depths of seawater (surface, middle, and bottom) and sediment in YRE. Panels **(A,B)** represent the Chao1 and Shannon indices of different samples, respectively; Panel **(C)** represents the Spearman correlation coefficient between the environmental factors and the alpha-diversity indices. The circle sizes in heatmaps represent the importance of the environmental factors on the alpha-diversity indices based on the multiple linear regression and variance decomposition analysis. The bar chart represents the explanation degrees in the multiple linear regression of the environmental factors and the alpha-diversity indices. The symbols “*”, “**”, “***”, and “****” represent 0.01 ≤ *p* < 0.05, 0.005 ≤ *p* < 0.01, 0.001 ≤ *p* < 0.005, and *p* < 0.001, respectively.

The alpha-diversity index of Chao1 was not significantly different among various layers of seawater and sediment (ANOVA, *p* > 0.05; Figure 3A), except that the Chao1 index of the middle layer was significantly lower than the bottom layer of seawater (ANOVA, *p* < 0.05; Figure 3A). The Shannon index of sediment was significantly higher than different layers of seawater (ANOVA, *p* < 0.01; Figure 3B). The Shannon index of the middle layer was significantly lower than the bottom layer of seawater (ANOVA, *p* < 0.01; Figure 3B), and no significant difference was found among other seawater layers (ANOVA, *p* > 0.05; Figure 3B). The results showed that NH_4_-N was positively correlated with abundance indices (Richness, ACE, and Chao1) and diversity indices (Shannon and Simpson). In addition, NH_4_-N had a higher effect on the abundance index but had little effect on the diversity index (Figure 3C).

**Figure 4:**
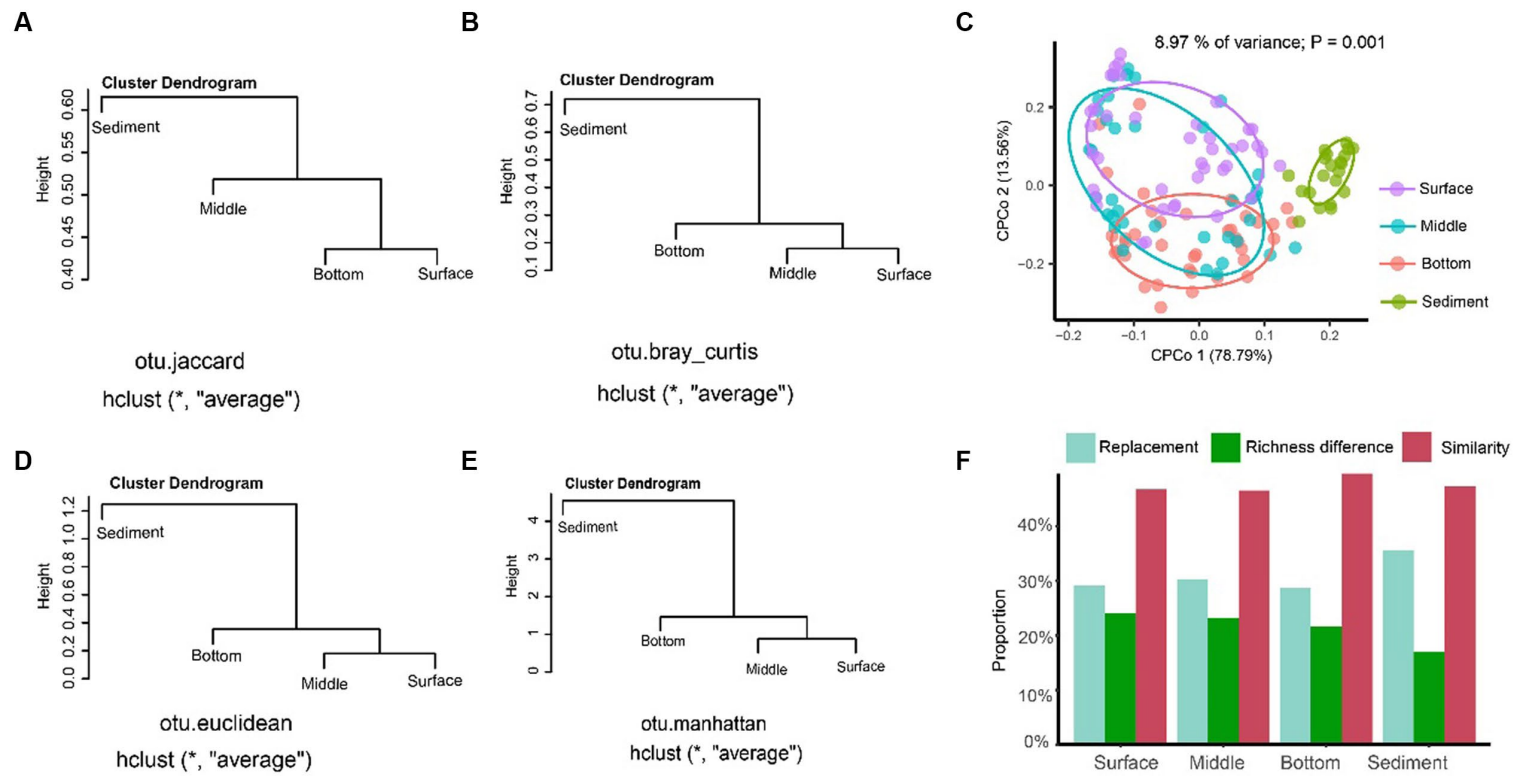
The beta-diversity analysis of the fungal communities in YRE. Panels **(A–D)** represent the results of the unweighted pair-group method with arithmetic means (UPGMA) clustering analysis based on the Jaccard, Bray-Curtis, Euclidean, and Manhattan distances, respectively. Panel **(E)** is the constrained principal coordinate analysis (CPCoA) based on the Bray-Curtis distance of the fungal communities from the seawater and sediment samples. Panel **(F)** is the result of the proportion of the replacement and richness difference components of fungal community dissimilarity from the seawater and sediment samples.

The result of UPGMA showed that the sediment could be separated from the seawater samples of different layers regardless of distance algorithms including Jaccard (Figure 4A), Bray–Curtis (Figure 4B), Euclidean (Figure 4C), and Manhattan distances (Figure 4D). The CPCoA result showed that only the fungal communities in the sediment samples were significantly separated from the seawater samples, and the seawater samples with different depths were not separated according to the 95% confidence ellipse, as shown in Figure 4E. The beta-diversity partitioning results showed that the species replacement was the main contributor for the community composition differences, which contributed 54.72% (surface layer), 56.61% (middle layer), 56.95% (bottom layer), and 67.60% (sediment) for the samples, respectively. However, the contribution of richness difference to the beta-diversity dissimilarity was relatively low, which were 45.18% (surface layer), 43.38% (middle layer), 43.05% (bottom layer), and 32.40% (sediment), respectively (Figure 4F).

### Influence of environmental variables on the structure of fungal communities in YRE

**Figure 5:**
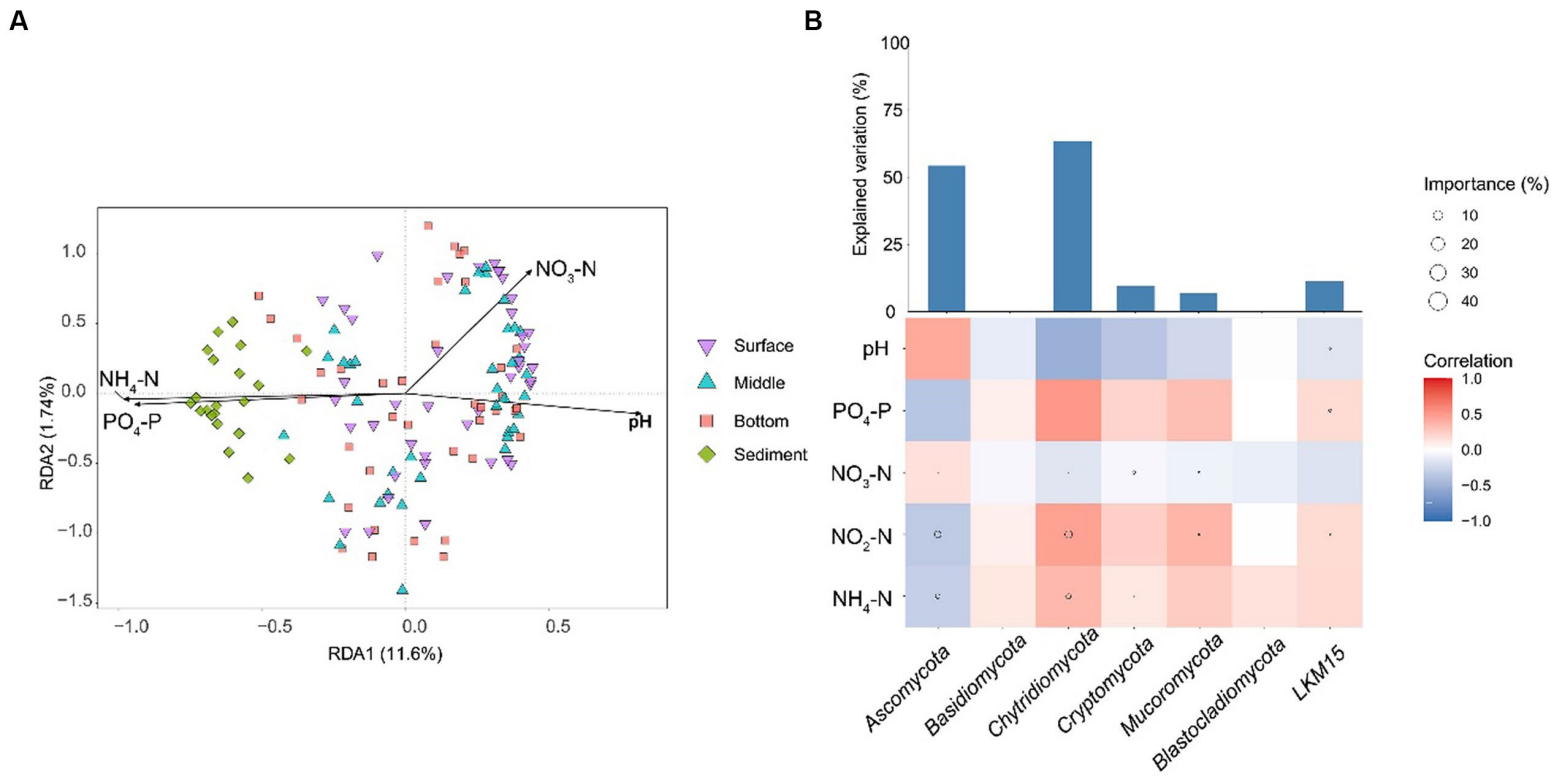
**(A)** db-RDA displays the relationship between fungal community structures and environmental factors. **(B)** The influence of environmental factors on the relative abundances of fungal phyla. The heatmap represents the Spearman correlation coefficient between the environmental factors and the relative abundances of fungal phyla. The circle size in heatmaps represents the importance of the environmental factors on the relative abundances of fungal phyla based on the multiple linear regression and variance decomposition analysis. The bar chart represents the explanation degrees in the multiple linear regression of the environmental factors and the relative abundances of fungal phyla. NH_4_-N, ammonia nitrogen; NO_2_-N, nitrite nitrogen; PO_4_-P, phosphorus; NO_3_-N, nitrate.

Owing to that the gradient length along the axis was less than 4 in the results of DCA, db-RDA was selected to describe the relationship between environmental factors and fungal communities. The results showed that all the environmental factors had significant effects on fungal communities (*p* < 0.001; Figure 5A). NH_4_-N was the most influential environmental factor (*p* < 0.001; R^2^ = 0.5169) for the fungal community structure (Supplementary Table S2). The NH_4_-N was negatively correlated with the relative abundance of Ascomycota and was positively correlated with the relative abundances of other phyla (Figure 5B).

### Distance–decay pattern analysis

**Figure 6:**
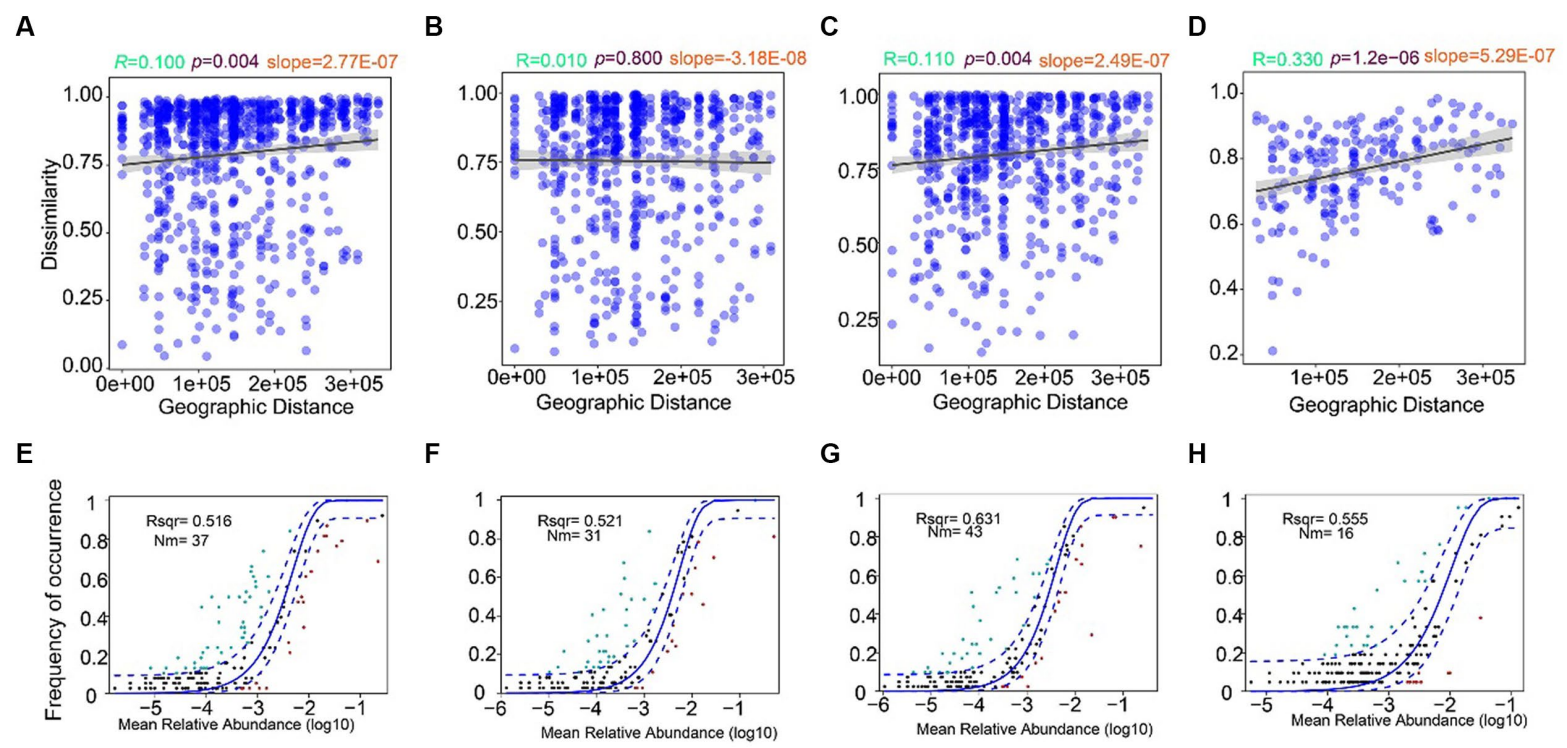
Distance–decay patterns of the fungal communities in YRE. Panels **(A–D)** represent the distance–decay curves of dissimilarity for the fungal communities in YRE from surface, middle, bottom, and sediment samples, respectively. Panels **(E–H)** represent the fit of the neutral community model (NCM) of fungal community assembly in the surface, middle, bottom, and sediment samples, respectively. The solid blue lines indicate the best fit to the NCM as in Sloan et al. (2006), and the dashed blue lines represent the 95% confidence intervals in the model prediction. ASVs that occurred more and less frequently than the predicted values in the NCM are shown in green and red, respectively. Nm indicates the products of metacommunity size and migration rate; Rsqr indicates the fit goodness of this model.

The distance–decay patterns of the fungal communities in seawater (Figures 6A–C) and sediment (Figure 6D) samples were analyzed. A significant and negative distance–decay pattern was found in the samples from the surface (Figure 6A), bottom (Figure 6B) seawater, and sediment (Figure 6D; *p* < 0.01); however, the distance–decay pattern was not significant in the middle seawater (Figure 6C). In addition, the slope of the sediment’s community dissimilarity (slope = 5.29E-07) was higher than those of the seawater samples, indicating the spatial turnover rate of the fungal communities of sediment was higher than the seawater. The slope of seawater from the surface layer (slope = 2.77E-07) was higher than the middle (slope = 3.18E-08) and bottom layers (slope = 2.49E-07). Hence, the dispersal of fungi community in the surface layer was more restricted than that in other layers in the seawater of YRE (Figures 6A–D).

### The NCM analysis of the fungal communities in YRE

The NCM was used to predict the relationship between the occurrence frequency of ASV and its relative abundance in fungal communities from the different layers of seawater and sediment (Figures 6E–H). The relative contribution of stochastic processes increased gradually with the water depth, explaining 51.6% (Figure 6E), 52.1% (Figure 6F), and 63.1% (Figure 6G) of the fungal community variation in the surface, middle, and bottom layers of seawater, respectively. The explained community variation of fungal community in sediment was 55.5% (Figure 6H), thereby the effect of the stochastic process in sediment was lower than in bottom seawater but higher than in the surface and middle seawater. Furthermore, the Nm value of sediment (16) was lower than those of the seawater from different depths (surface = 37, middle = 31, and bottom = 43). These results indicated that dispersal limitation was more dominant in the fungal community assembly processes in the sediment than in the seawater samples, and the dispersal limitation was more dominant in the fungal community assembly processes in the middle layer seawater than in the surface and bottom layers.

### The null model analysis of the fungal communities in YRE

**Figure 7:**
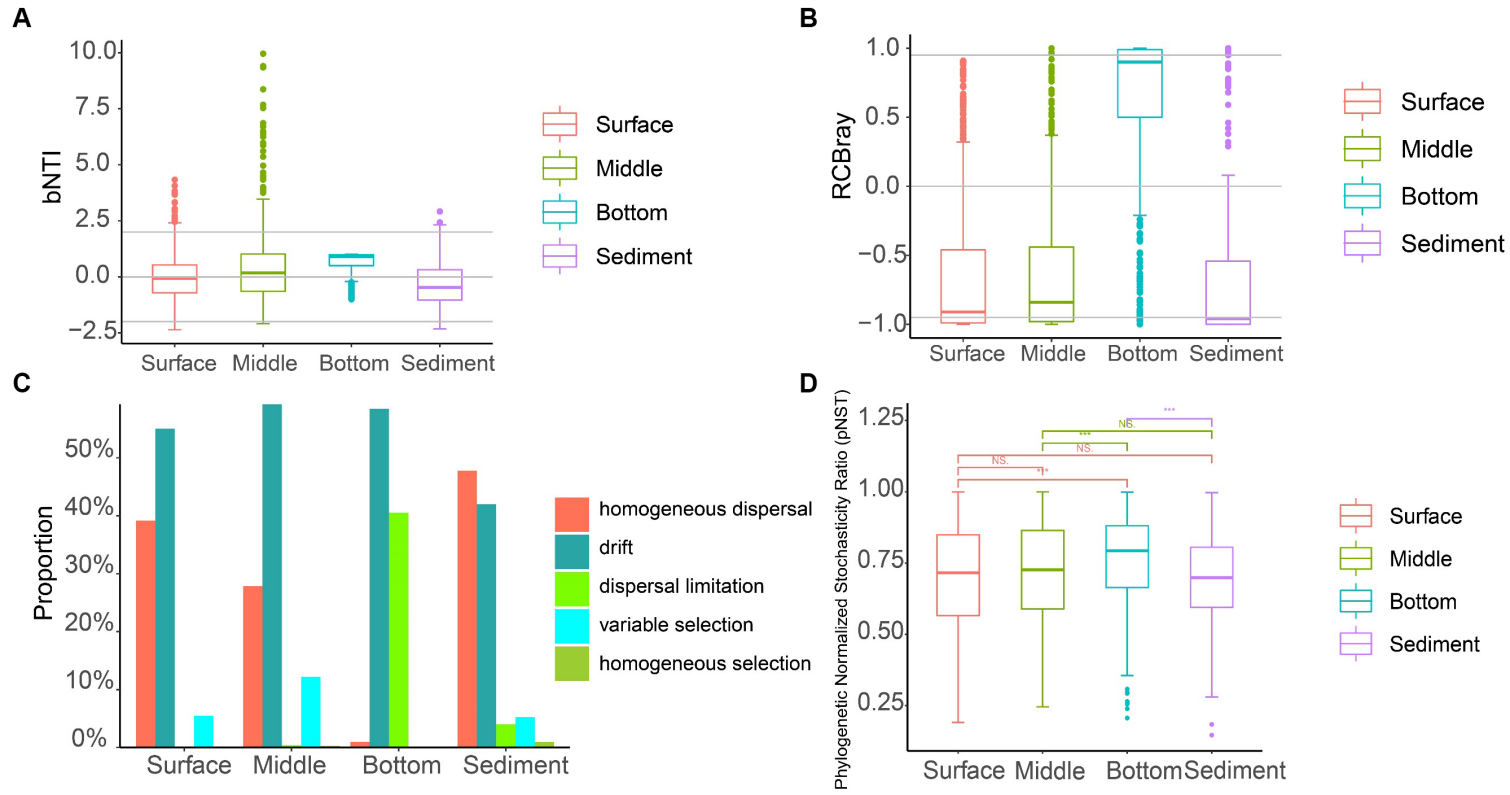
The null model analysis of fungal communities in the seawater and sediment samples of YRE. **(A)** Boxplots of the beta nearest-taxon index (βNTI) in the seawater and sediment samples. Gray lines represent βNTI values ranging from 2 to −2. **(B)** Bray–Curtis-based Raup-Crick (RCBray) values range from 0.95 to −0.95. **(C)** The contribution rate of deterministic processes (homogeneous and variable selection) and stochastic processes (homogeneous dispersal, drift, and dispersal limitation) to the fungal community assembly. **(D)** The phylogenetic normalized stochasticity ratio (pNST) distribution of fungal communities in seawater and sediment samples. “***” indicates *p* < 0.001.

The null model analysis showed that the βNTI values in all the samples were between −2 and 2, suggesting that stochastic processes were more important than deterministic processes (Figure 7A). Most of the RCBray values ranged from −0.95 to 0.95 (Figure 7B), indicating the drift was the significant player for the assembly process of the fungal community. The drift (55.02%), being the predominant stochastic process, dominated the fungal community assembly in the seawater and sediment samples (Figure 7C). The pNST was used to quantify the relative importance of deterministic and stochastic processes in community assembly. The results showed that the pNST values of fungal communities in different groups were mainly distributed in the interval > 0.5 (Figure 7D), showing that the stochastic processes were dominant in this fungal community assembly.

### Co-occurrence network analysis

**Figure 8:**
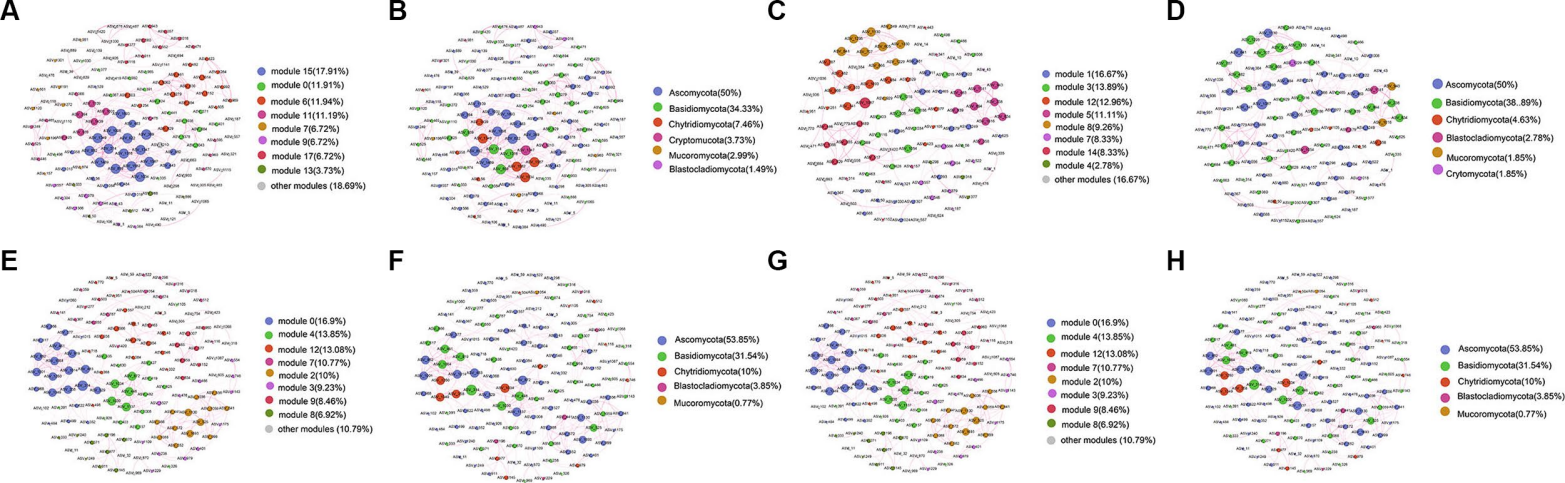
Co-occurrence network analysis of fungal communities from the surface **(A,B)**, middle **(C,D)**, bottom **(E,F)**, and sediment **(G,H)** samples in YRE. The nodes from the networks in **(A,C,E,G)** were colored based on the modularity class, and the nodes from the networks **(B,D,F,H)** were colored based on the fungal phyla. The node size was directly proportional to the degree values of the networks, and edge thickness depended on the correlation among the taxa. A positive correlation is displayed with a purple line.

A co-occurrence network consisting of 458 nodes and 134 edges was generated for the surface layer of the seawater (Figure 8A). The nodes in the network were assigned into 6 phyla (Figure 8B). The middle layer network contained 16 modules (Figure 8C), and the nodes in the network were assigned into 6 phyla (Figure 8D). The bottom layer network contained 14 modules (Figure 8E), and the nodes in the network were assigned into 5 phyla (Figure 8F). The sediment network contained 8 modules (Figure 8G), and the nodes in the network were assigned into 7 phyla (Figure 8H). The relative abundances of Ascomycota and Basidiomycota were higher than others in all networks (Figures 8B,D,F,H). The average clustering coefficient of the sediment network was higher than that of the seawater networks, indicating that ASVs from the sediment samples were more closely related to each other (Supplementary Table S3). The topological properties are summarized in Supplementary Table S3.

The Zi-Pi plot showed that 30, 26, 27, and 37 Ascomycota ASVs served as the calculated keystone taxa in the fungal networks of surface (Supplementary Figure S2A), middle (Supplementary Figure S2B), bottom (Supplementary Figure S2C), and sediment (Supplementary Figure S2D) samples. The community composition of the keystone taxa in different groups (Supplementary Figure S3) showed that the relative abundance of Ascomycota was higher in sediment than those in surface, middle, and bottom samples. Basidiomycota with high relative abundances and Mucoromycota with low relative abundances was identified as the calculated keystone taxon.

## Discussion

### Species replacement and environmental variables served as the contributors to the fungal community variation in YRE

The current study found that there was no significant difference in the relative abundances of fungi within the collected samples, but the species diversity index (Shannon) was significantly (*p* < 0.05) higher in sediments than the seawater from different layers. This is consistent with a previous study on the Yellow River Estuary (Shi et al., 2020). However, other studies have obtained different results. For example, the diversity and abundance of fungal communities in the Western Pacific (Li et al., 2019) are obviously different in various water layers, and the fungal communities in the Bohai Sea and the Yellow Sea showed regional differences in the seawater (Gong et al., 2015; Wang Y. et al., 2017). The dispute could be probably attributed to the different sampling locations and the effect of different ocean currents on local ocean formations.

Previous studies have shown that the total β-diversity was formed by species replacement and richness difference (Baselga, 2010; Legendre, 2014; Wang et al., 2015). The species replacement, also known as turnover, reflects the species turnover along spatial or environmental gradients (Legendre, 2014), while the richness difference, known as species nestedness, represents the non-random process of species gain or loss (Baselga, 2010; Baselga, 2013). Species replacement explained the higher rate of β-diversity than richness difference in this study, implying that the β-diversity of fungal communities was mainly derived from species turnover components in the YRE. This is consistent with the previous reports on the typical dryland ecosystem of northwest China (Wang J. et al., 2017) and soil fungal communities (Liu W. et al., 2022).

Among the environmental factors collected in the current study, NH_4_-N mostly shaped the fungal community and impacted the phylum relative abundances, including Ascomycota, Chytridiomycota, and Cryptomycota in the samples. Zhen et al. (2017) reported that nitrogen is essential for life activities as a direct or indirect part. In the marine environment, nitrogen is an essential nutrient element for primary production and nitrogen bioavailability, thereby shaping the plankton diversity and biological processes (Gruber and Galloway, 2008; Wannicke et al., 2018). The marine environment is typically nitrogen deficient (Zhang Y. et al., 2016). Therefore, NH_4_-N could contribute to and be of value to the growth of fungal communities in the ocean. Previous studies have shown that planktonic fungal diversity and ASV abundance are largely regulated by the changes in the availability of several potential growth substrates, such as organic and inorganic nitrogen-rich substrates (Taylor and Cunliffe, 2016).

### Stochastic processes dominated the fungal community assembly in YRE

Clarifying the community assembly process is ecologically crucial for understanding the adaptability of the microorganisms to the environmental variation in water-related ecosystems (Zhou and Ning, 2017). In the limited studies on the assembly process of fungal communities, the stochastic process has been demonstrated as the dominant process that drives the fungal communities in mangrove sediment (Zhang et al., 2021), smelting soils (Liu B. et al., 2022), estuarine wetlands (Huang L. et al., 2022), and coastal line (Zhao et al., 2023). Nevertheless, the detailed components, including homogenizing dispersal, dispersal limitation, and drift, in the stochastic processes are rarely assessed in the assembly of fungal communities (Zhou and Ning, 2017).

The null model analysis shows that the stochastic process plays an important role in assembling the fungal community in the YRE. More specifically, the drift in the stochastic process dominated the fungal community assembly in the seawater samples of YRE, and homogeneous dispersal in the stochastic process mainly shaped the sediment fungal communities. Ecological drift is a central concept in community ecology, which is the random change in the relative abundance of different species within a community over time to species identity due to the inherent stochastic processes of birth, death, and reproduction (Nemergut et al., 2013a; Vellend et al., 2014). A study has shown that random births and deaths are more important in shaping communities with smaller population sizes (Samad et al., 2017); therefore, the weak selection and small size community of fungi probably resulted in the increasing importance of drift for this community assembly (Chase and Myers, 2011).

The dispersal limitation also functioned for the fungal community assembly based on the distance–decay model, whose result in current study indicated that (i) the fungal communities in the YRE had spatial structure distribution patterns; (ii) fungal communities in sediment and surface seawater were more controlled by dispersal limitation due to their higher slopes; (iii) and the fungal communities in middle seawater were affected by not only dispersal limitation but also the spatial environment due to its non-significance in the model. The stronger dispersal limitation has been demonstrated in sediment fungal communities because of the microbial colonization pattern (Shurin et al., 2009; Zhao et al., 2022). Compared with other water layers, microorganisms in surface seawater dispersed more easily due to their small size and inability to counteract the flow of unidirectionality and ocean currents (Chen et al., 2019). In addition, we primarily attributed the high dispersal limitation for the fungal communities in the surface seawater to the ocean current conditions in YRE. Yangtze River diluted water, Taiwan Strait, and other currents formed a complex salt font environment between the fresh plume and salty water in YRE (Wei et al., 2007), thereby potentially limiting the diffusion of fungal communities. Similar results were obtained from other estuaries, such as the Yellow River Estuary (Wang et al., 2021) and the Pearl River Estuary (Lu et al., 2022).

In general, the endogenous (community size) and exogenous (current environment) factors were the potential reasons that increased the significance of stochastic processes for the assembly of fungal community in YRE, indicating that the changes and undulation of these factors in YRE, such as acidification and seasonal hypoxia (Lyu et al., 2022), could obviously vary the fungal communities and their ecological functions.

### Keystone taxa of the fungal communities of YRE

The interactions and keystone taxa of fungal communities in YRE have scarcely been reported in former studies. The current study showed that the fungal network structure in the sediment is more complex than those in the seawater from various layers. Previous studies have found that low bacterial diversity reduces the complexity of symbiotic networks in mountain ecosystems (Li et al., 2020) and mangrove ecosystems (Chen and Wen, 2021). Therefore, the higher fungal Shannon index of sediment samples in YRE might result in more complex species interactions. In addition, studies have shown that eukaryotic plankton symbiosis networks are influenced by different environmental factors due to their various influences (i.e., pH and total nitrogen; Liu et al., 2019). Thus, the different effects of the environmental factors collected in this study on the fungal communities could be another reason for the high network complexity of the sediment samples.

The average clustering coefficients of the fungal networks in sediment samples were higher than those of seawater samples from different layers, and the average path lengths of sediment samples were lower than those of seawater samples, indicating that the fungal species in sediments were related more closely than the water samples, which could be the consequence of the sediment fungi being controlled by dispersal limitation. The higher diversity and closer interconnection could give the fungal communities in sediments a stronger buffer against environmental disturbances. In all the networks, the proportion of positive correlation was higher than the negative correlation, which reveals that positive effects (i.e., reciprocity and/or homology, where two species exchange metabolites in favor of both) were more important than negative effects (i.e., predator–prey relationships, host–parasite relationships, and/or competition between microbes; Chen and Wen, 2021). This is similar to the result of global ocean plankton interactions conducted by the Tara Oceans Project (Lima-Mendez et al., 2015) and fungal investigation in mangroves (Zhang et al., 2021; Zuo et al., 2022), indicating that fungi tend to benefit each other in these marine environments.

Microbial co-occurrence network generally can be divided into several connected modules. These modules may reflect the habitat heterogeneity, system development close related species, ecological niche overlap, and species evolution, which is regarded as a system development, evolution, or functionally independent unit (Olesen et al., 2007). The key nodes identified in ecological network modules often represent key species that may play an important role in maintaining the stability of microbial community structure (Shi et al., 2016). However, the keystone fungal taxa remained unclear for the estuarine ecosystems. Ascomycota and Basidiomycota with high relative abundances were identified as the keystone fungal taxa in YRE, which was consistent with the former fungal study on Jinsha River (Chen et al., 2020). Mucoromycota with low relative abundances was also identified as a keystone taxon. Increasing evidence from different habitats indicates the importance of rare and less abundant species in microbial networks (Xue et al., 2018), and their removal can lead to dramatic changes in microbiome structure and function (Banerjee et al., 2018). Therefore, more attention should be paid to the detailed taxonomic information and maintenance function of these taxa in future studies on the YRE ecosystem functions.

In conclusion, the present study proved that the fungal communities in the YRE had a typical distance–decay pattern, and the stochastic process dominated the assembly of fungal communities in the YRE. This study points out the importance of drift and homogeneous dispersal for the fungal community structures in seawater and sediment samples from YRE, respectively, which enhances our understanding of fungal community aggregation and interaction and facilitates the future study of the fungal ecological functions in the YRE ecosystem.

## Data availability statement

Raw sequence data are deposited at the NCBI Sequence Read Archive database (https://www.ncbi.nlm.nih.gov/sra) under the accession numbers of SRR21079128- SRR21079264.

## Ethics statement

No animal studies are presented in this manuscript.

## Author contributions

WQ and YZu wrote the manuscript. YZu and YZh conducted the experiments and analyzed the data. WQ and JW conceived, designed, and financially supported the research. YZu and WQ analyze the data. WQ, JW, and YZu revised the manuscript. All authors contributed to the article and approved the submitted version.
