# Supplementary material for: Structure and assembly process of fungal communities in the Yangtze River Estuary
Source: Front Microbiol. 2024 Jan 8;14:1220239. doi: 10.3389/fmicb.2023.1220239 (PMC10800840; doi:10.3389/fmicb.2023.1220239)
Supplement: Supplementary file 1 [file Data_Sheet_1.pdf]

Structure and assembly process of fungal communities in the Yangtze River Estuary

Wu Qu [1#], Yaqiang Zuo[1#], Yixuan Zhang[1], Jianxin Wang [1*]

1. Marine Science and Technical College, Zhejiang Ocean University, Zhoushan, China

#These authors contribute equally to this work

*Corresponding author: Jianxin Wang

Corresponding author e-mail: jxwang@zjou.edu.cn

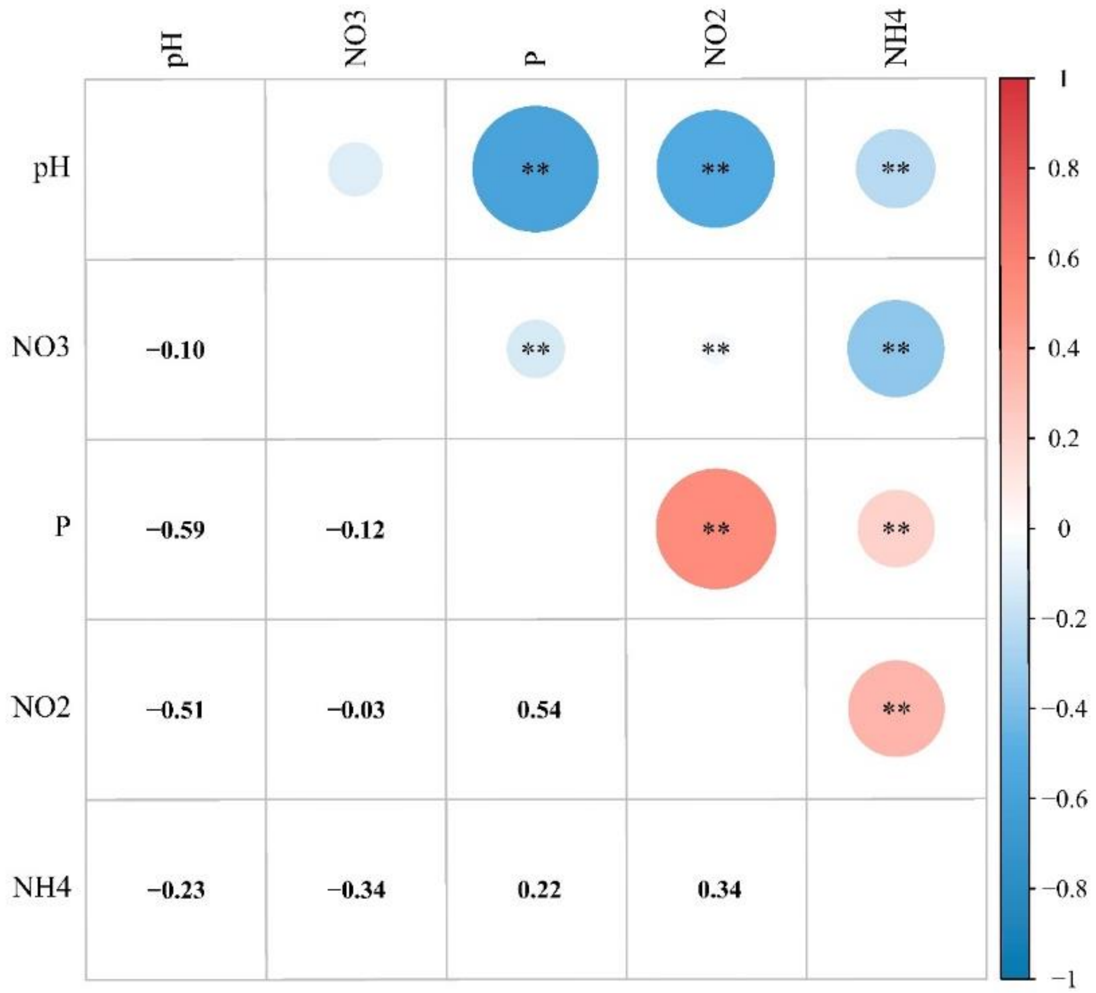

Fig. S1. The Pearson correlations among the environmental factors. The correlation coefficient was shown in the matrix at the lower left. "**", $p<0.01$. NH4, the ammonia nitrogen; NO2, nitrite nitrogen; P , phosphorus; NO3, nitrate.

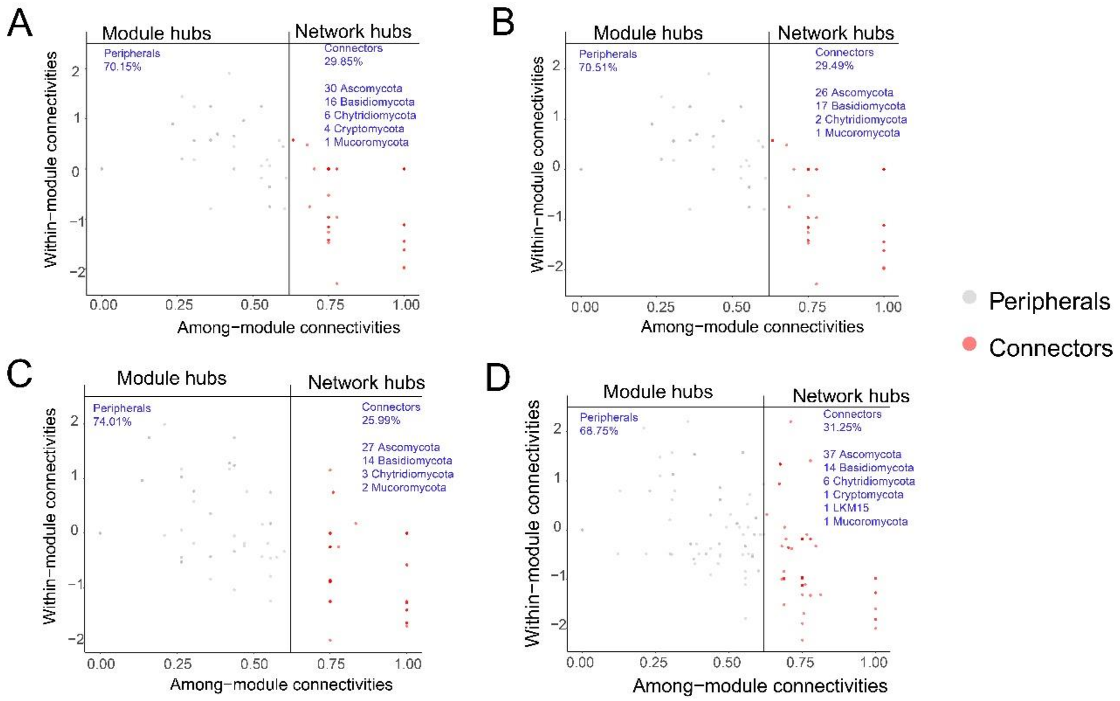

Fig. S2. Zi-Pi plot showing the distribution of fungal ASVs based on their topological roles. Each symbol represents an ASV. The topological role of each ASV was determined according to the scatter plot of within-module connectivity (Zi) and among-module connectivity (Pi). (i) network hubs: nodes with Zi > 2.5 and Pi > 0.62; (ii) module hubs: nodes with Zi > 2.5 and Pi ≤0.62; (iii) connectors: nodes with Zi≤2.5 and Pi > 0.62; and (iv) peripheral nodes: nodes with Zi≤2.5 and Pi ≤0.62.

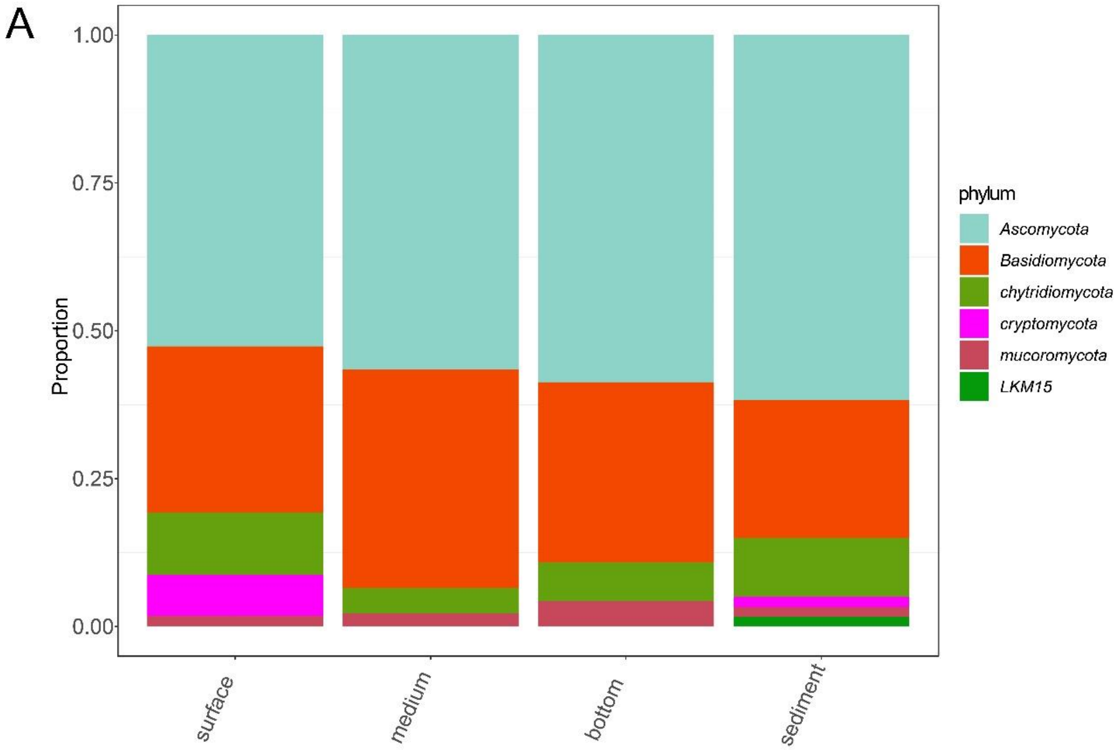

Fig. S3. The stacked bar plot showed the proportion of the fungal community composition of the keystone taxa in different groups.

Table S1. Environmental properties of the seawater and sediments in the Yangtze River Estuary.

Sample	pH	P（mg/L）	NH4（mg/L）	NO3（μmol/L）	NO2（mg/L）
M0	8.08	3.63	5.16	1.05	0.78
M0BP	7.94	0.0089	0.0288	29.3908	0.0044
M0SF	7.95	0.0387	0.024	35.1854	0.0061
M0SP	7.95	0.0387	0.024	35.1854	0.0061
M1	7.14	2.83	10.07	1.25	0.78
M1BF	7.88	0.0152	0.0277	13.0295	0.0146
M1BP	7.88	0.0152	0.0277	13.0295	0.0146
M1MF	7.92	0.0294	0.0258	28.6723	0.0102
M1MP	7.92	0.0294	0.0258	28.6723	0.0102
M1SF	7.93	0.0602	0.0267	30.9122	0.0094
M1SP	7.93	0.0602	0.0267	30.9122	0.0094
M2	7.68	2.52	9.07	1.3	0.82
M2BF	7.97	0.0095	0.0275	8.3143	0.0027
M2BP	7.97	0.0095	0.0275	8.3143	0.0027
M2MF	7.99	0.0083	0.0279	7.5758	0.0033
M2MP	7.99	0.0083	0.0279	7.5758	0.0033
M2SF	8.28	0	0.0309	5.417	0.0044
M2SP	8.28	0	0.0309	5.417	0.0044
N0	7.51	1.43	10.11	1.4	0.78
N0BF	7.81	0.0379	0.0184	36.6905	0.0181
N0BP	7.81	0.0379	0.0184	36.6905	0.0181
N0SF	7.91	0.0254	0.0055	54.6433	0.0097
N0SP	7.91	0.0254	0.0055	54.6433	0.0097
N1	7.43	2.36	9.61	1.45	0.74
N1BP	7.84	0.016	0.025	14.1657	0.0193
N1MF	7.84	0.0126	0.0223	14.0535	0.0131
N1MP	7.84	0.0126	0.0223	14.0535	0.0131
N1SF	7.92	0.0162	0.0089	41.8519	0.0062
N1SP	7.92	0.0162	0.0089	41.8519	0.0062
N2	7.1	1.15	9.11	1.5	0.82
N2BF	7.84	0.0108	0.0256	4.7966	0.0139
N2BP	7.84	0.0108	0.0256	4.7966	0.0139
N2MF	7.85	0.0114	0.0218	5.6943	0.0112
N2MP	7.85	0.0114	0.0218	5.6943	0.0112
N2SF	8.2	0	0.0271	9.0604	0.012
N2SP	8.2	0	0.0271	9.0604	0.012
N3	7.85	1.42	7.15	1.25	0.78
N3BF	7.9	0.0237	0.0289	7.9853	0.006
N3BP	7.9	0.0237	0.0289	7.9853	0.006
N3MF	7.91	0.0072	0.0324	5.6382	0.0136
N3MP	7.91	0.0072	0.0324	5.6382	0.0136
N3SF	8.46	0	0.0306	4.5161	0.0089

N3SP	8.46	0	0.0306	4.5161	0.0089
N4	7.16	1.1	2.55	1.3	0.77
N4BF	7.91	0.0216	0.0292	5.1893	0.0046
N4MF	7.94	0.0122	0.0284	5.5468	0.0058
N4MP	7.94	0.0122	0.0284	5.5468	0.0058
N4SF	8.42	0	0.0282	5.4138	0.0087
N4SP	8.42	0	0.0282	5.4138	0.0087
N5	7.16	3.22	5.12	0.95	0.74
N5BF	7.93	0.0062	0.0216	6.1431	0.0017
N5BP	7.93	0.0062	0.0216	6.1431	0.0017
N5MF	7.98	0.003	0.0248	4.1379	0.0024
N5MP	7.98	0.003	0.0248	4.1379	0.0024
N5SP	8.29	8.00E-04	0.0275	4.6785	0.0037
N6	7.11	3.29	5.62	1	0.67
N6BF	7.94	0.0072	0.0293	4.3547	0.0043
N6BP	7.94	0.0072	0.0293	4.3547	0.0043
N6MF	7.94	0.0043	0.0233	4.6256	0.004
N6MP	7.94	0.0043	0.0233	4.6256	0.004
N6SF	8.34	0	0.0254	2.265	0.0061
N6SP	8.34	0	0.0254	2.265	0.0061
N7	7.23	3.36	4.56	1.05	0.94
N7BF	7.93	0.0045	0.0185	5.0591	0.0022
N7BP	7.93	0.0045	0.0185	5.0591	0.0022
N7MF	7.94	0.0064	0.0195	3.7586	0.0015
N7MP	7.94	0.0064	0.0195	3.7586	0.0015
N7SF	8.26	5.00E-04	0.0265	3.1058	0.0037
N7SP	8.26	5.00E-04	0.0265	3.1058	0.0037
P1	7.05	1.89	3.11	1.5	0.67
P1BF	7.94	0.0245	0.0262	11.7797	0.0062
P1BP	7.94	0.0245	0.0262	11.7797	0.0062
P1MF	8.13	0	0.0276	5.3602	0.0117
P1MP	8.13	0	0.0276	5.3602	0.0117
P1SF	8.24	0	0.0289	4.3376	0
P1SP	8.24	0	0.0289	4.3376	0
P2	6.82	3.65	4.56	1.55	0.63
P2BF	7.87	0.0103	0.0345	11.3045	0.0037
P2BP	7.87	0.0103	0.0345	11.3045	0.0037
P2MF	7.9	0.0049	0.0215	13.4364	0.0158
P2MP	7.9	0.0049	0.0215	13.4364	0.0158
P2SF	8.02	0.0099	0.0227	22.5368	0.0189
P2SP	8.02	0.0099	0.0227	22.5368	0.0189
S1	7.21	1.62	4.58	1.05	0.67
S1BF	7.9	0.0281	0.0189	9.6651	0.0147
S1BP	7.9	0.0281	0.0189	9.6651	0.0147

S1MF	7.97	0.0291	0.0155	15.0296	0.0122
S1MP	7.97	0.0291	0.0155	15.0296	0.0122
S1SF	7.96	0.0331	0.0227	14.0001	0.009
S1SP	7.96	0.0331	0.0227	14.0001	0.009
S2	7.63	3.68	4.06	1.5	0.7
S2BF	7.98	0.0362	0.0156	5.1133	0.0043
S2BP	7.98	0.0362	0.0156	5.1133	0.0043
S2MF	7.97	0.0066	0.0199	2.7877	0.0082
S2MP	7.97	0.0066	0.0199	2.7877	0.0082
S2SF	8.22	0.0076	0.0188	2.106	0.0392
S2SP	8.22	0.0076	0.0188	2.106	0.0392
S3	7.17	1.02	4.11	1.25	0.78
S3BF	8.02	0.0166	0.0241	3.867	0.0058
S3BP	8.02	0.0166	0.0241	3.867	0.0058
S3MF	8.14	0.0081	0.0165	0.6699	0.0027
S3MP	8.14	0.0081	0.0165	0.6699	0.0027
S3SF	8.17	0.0051	0.0237	0.5074	9.00E-04
S3SP	8.17	0.0051	0.0237	0.5074	9.00E-04
S4	8.12	1.22	3.57	1.35	0.67
S4BF	7.94	0.0638	0.0251	6.4138	0.004
S4BP	7.94	0.0638	0.0251	6.4138	0.004
S4MF	8	0.0876	0.0266	3.2709	0.012
S4MP	8	0.0876	0.0266	3.2709	0.012
S4SF	8.23	0.7427	0.0284	2.3232	0.0067
S4SP	8.23	0.7427	0.0284	2.3232	0.0067
S5	7.62	1.13	2.51	1.3	0.63
S5BF	7.94	0.0273	0.0231	7.335	0.004
S5BP	7.94	0.0273	0.0231	7.335	0.004
S5MF	7.97	0.0222	0.0345	6.0887	0.013
S5MP	7.97	0.0222	0.0345	6.0887	0.013
S5SF	8.28	0.0252	0.0201	3.4335	0.01
S5SP	8.28	0.0252	0.0201	3.4335	0.01
S6	7.81	1.88	3.5	1.2	0.78
S6BF	7.94	0.0212	0.0333	6.0345	0.0029
S6BP	7.94	0.0212	0.0333	6.0345	0.0029
S6MF	8.22	0.0245	0.0175	1.0118	0.0018
S6MP	8.22	0.0245	0.0175	1.0118	0.0018
S6SF	8.26	0.0168	0.0191	1.5754	0.003
S6SP	8.26	0.0168	0.0191	1.5754	0.003
S7	7.56	1.52	5.11	1.5	0.82
S7BF	7.98	0.0168	0.0281	5.8719	0.0035
S7BP	7.98	0.0168	0.0281	5.8719	0.0035
S7MP	8.14	0.0035	0.0264	0.3291	5.00E-04
S7SF	8.21	0.0033	0.0306	1.5971	0.0028

S7SP	8.21	0.0033	0.0306	1.5971	0.0028
Z	7.89	3.56	10.1	1.4	0.43
ZBP	7.95	0.0085	0.0257	8.2189	0.0062
ZMF	8.01	0.006	0.0278	9.0604	0.0091
ZMP	8.01	0.006	0.0278	9.0604	0.0091
ZSF	8.25	0	0.0318	7.6579	0.0084
ZSP	8.25	0	0.0318	7.6579	0.0084

Table S2. The result of distance-based redundancy analysis among fungal communities in Yangtze River Estuary.

	RDA1	RDA2	r^2	*p*
pH	0.98605	-0.16645	0.3844	0.001
P	-0.99763	0.06881	0.4738	0.001
NH4	-0.99637	0.08510	0.5169	0.001
NO3	0.75249	0.65861	0.2302	0.001

Table S3. Topological properties of co-occurring networks in different depths of sea water and sediment.

group	surface	medium	bottom	sediment
edge	134	108	130	170
node	458	231	354	1066
Modularity (MD)	0.593	0.763	0.759	0.47
Clustering coefficient (CC)	0.667	0.629	0.646	0.546
Average path length (APL)	4.512	4.975	7.008	3.612
Network diameter (ND)	14	11	18	11
Average degree (AD)	6.836	4.278	5.446	12.541
